# AUDIT-C as a possible source of referral during the COVID-19 pandemic for participants presenting patterns of high-risk alcohol consumption in a South African township

**DOI:** 10.1186/s12889-023-16775-5

**Published:** 2023-09-30

**Authors:** Lynne Goldschmidt, Buyisile Mncina, Malose Langa, Steven Rebello, Thokozile Budaza, Josephine Tshabalala, Yohannes Kinfu, Tom Achoki

**Affiliations:** 1 Department of Psychology, University of the Witwatersrand, Johannesburg, South Africa; 2 Centre for the Study of Violence and Reconciliation, Johannesburg, South Africa; 3 Private Practice, Johannesburg, South Africa; 4 ABInBev Foundation, New York, NY USA; 5 Department of Health Metrics Sciences, University of Washington, Seattle, USA; 6 United Nations University, International Institute for Global Health (UNU-IIGH), Kuala Lumpur, Malaysia; 7 Africa Institute for Health Policy, Nairobi, Kenya

**Keywords:** AUDIT-C, Alcohol consumption, Lay counsellors, Low socioeconomic setting, Unhealthy alcohol use, Prevention

## Abstract

**Background:**

Unhealthy alcohol use is a leading contributor to premature death and disability worldwide. The World Health Organization’s *Global Status Report on Alcohol and Health* ranked South Africa as having one of the riskiest patterns of alcohol consumption, which calls for intervention. Recognising the need for effective primary care interventions, particularly in the absence of appropriate alcohol-related harm reduction policies at national and local levels, this paper highlights the opportunities and challenges associated with a two-pronged, community-centred approach to the identification of unhealthy alcohol use and interventions.

**Methods:**

This approach included the use of the Alcohol Use Disorders Identification Test–Consumption (AUDIT-C) as a means of screening to identify individuals at moderate (score of 5–7) to high risk (score of 8 +) alcohol use, raising awareness, and investigating the potential utility of brief advice and referrals as a means of reducing risk.

**Results:**

Of the 54,187 participants, 43.0% reported engaging in moderate-risk alcohol consumption, with 22.1% reporting high-risk alcohol consumption. Resistance to brief advice was observed to increase with higher AUDIT-C scores. Similarly, participants engaging in high-risk alcohol consumption were resistant to accepting treatment referrals, with fewer than 10% open to receiving a referral.

**Conclusions:**

While men were most likely to report patterns of high-risk alcohol consumption, they were more resistant to accepting referrals. Additionally, participants who were willing to receive brief advice were often resistant to taking active steps to alter their alcohol use. This study highlights the need to consider how to prevent harmful patterns of alcohol use effectively and holistically, especially in low socioeconomic settings through primary health care and community services.

## Background

Unhealthy alcohol use is a significant cause of premature death and disability and has detrimental effects on overall societal wellbeing worldwide [1–3]. The widespread impact of alcohol use is of considerable concern, given the increased prevalence of consumption per capita over the last two decades [4, 5]. Although alcohol consumption is prevalent globally, significant regional differences are evident [2, 5]. Alcohol use in sub-Saharan Africa has continued to rise in recent years [5, 6], with South Africa being one of the countries with the highest alcohol consumption in the region. Alcohol consumption per person in South Africa is well above the African regional average [2, 6, 7]. Moreover, South Africa observes higher rates of binge drinking, which is defined as excessive alcohol use on a single occasion or within a short period of time [6].

The increasing prevalence of alcohol use highlights the urgent need for alcohol-related harm reduction policies and intervention strategies to reduce alcohol consumption. However, the South African public health system is overburdened, with little emphasis placed on interventions to reduce unhealthy alcohol use [8, 9]. The emergence of the COVID-19 pandemic highlighted this problem with the banning of alcohol sales to manage the concomitant rise in alcohol-related injuries, putting added pressure on the South African healthcare system [10].

An increased emphasis on reducing unhealthy alcohol use is likely to reduce the burden of preventable diseases and costs to public health systems [2, 7]. This highlights the need for effective alcohol-control policies and prevention and treatment strategies that address alcohol-related risks and harm. It has, however, been suggested that effective policy changes to address the risks presented by alcohol have consistently been subject to the lobbying pressures of the influential alcohol industry [9–11].

In response to the absence of adequate alcohol-related harm reduction policies, South Africa is therefore reliant on interventions that can be applied optimally within clinical settings and without burdening resources in terms of time and costs. However, various structural barriers in the South African context influence access to prevention and treatment interventions [12]. Structural barriers include the limited allocation of state resources to address unhealthy alcohol use, which hinder the state’s capacity to provide services [11, 12]. This is particularly relevant for those residing in historically disadvantaged South African townships [11, 12].

Globally, screening and brief educational and referral services have improved access to critical interventions [13, 14] and have proven to be efficient and cost-effective in addressing unhealthy alcohol use [15, 16]. Conversely, a recent meta-analysis suggested that it was not conclusive that brief alcohol interventions were effective in increasing the use of intervention services [17]. The research further called into question the effectiveness of referral to treatment as part of an alcohol screening and brief intervention effort. The aforementioned analysis was, however, premised on studies conducted in the Global North, which highlights the need for research that focuses on referral as a component of screening and brief interventions in the Global South.

This paper explores the efficacy of a screening and brief intervention in the South African context, focusing on four historically disadvantaged, resource-constrained sites in Alexandra township in Gauteng, South Africa. Specifically, the study aims to evaluate the applicability of the Alcohol Use Disorders Identification Test–Consumption (AUDIT-C) tool for identifying high-risk alcohol users and of subsequent brief intervention and, where needed, referral.

## Methods

### Study design

This research utilised a quantitative, cross-sectional, survey research design, in which participants were screened on one occasion utilising the AUDIT-C measure. Data collection occurred in Alexandra township, Johannesburg, South Africa, across all four quarters of 2021. The administration of the study coincided with the global COVID-19 pandemic and the national government’s consequent alcohol bans and travel restrictions. South Africa was one of the few countries to implement an alcohol ban intermittently as an emergency response [9].

The data collection process was managed by HIV South Africa (HIVSA), a not-for-profit organisation that has been in existence since 2002. HIVSA is a key strategic partner of the Gauteng Provincial Department of Health and the Gauteng Provincial Department of Social Development. Given HIVSA’s existing work in response to HIV and associated socioeconomic and health issues, the organisation was considered an appropriate partner to assist in the administration of the screening tool. HIVSA was responsible for recruiting, supervising and managing the lay counsellors who administered the AUDIT-C tool and offered brief intervention with or without referral. The lay counsellors received training over an initial three-day period followed by two additional one-day booster sessions. The training was facilitated by two registered counselling psychologists who were both experienced in providing brief intervention services. The training included the project’s aims, the study protocol, and the scoring of the AUDIT-C tool, followed by role-play exercises focusing on the brief advice and referral processes.

Data were collected in person using paper and pencil. The brief advice provided was premised on the Feedback, Responsibility, Advise, Menu for change, Empathy, and enhancing Self-efficacy (FRAMES) intervention framework [18], which focuses on feedback and advice components. Feedback comprised explaining the screening score to participants. Participants who received a low-risk score (AUDIT-C score of 1–4) were advised that while there is no completely safe level of alcohol use, their screening score suggested that they were drinking in a way that was less likely to result in harm to themselves or others. However, these participants were cautioned to keep track of their drinking to ensure that it did not gradually increase. Participants who received a moderate-risk score (AUDIT-C score of 5–7) were advised of the risk of experiencing harm from their drinking. Advice comprised explaining the best way to reduce the risk of harmful alcohol use, which focused on reducing the amount of alcohol consumed. Participants who received a high-risk score (AUDIT-C score of 8 +) were also advised that they were at risk of experiencing harm from their drinking. The AUDIT-C tool, in addition to the recommended intervention, took between 10 and 30 min to administer, depending on each participant’s score and the form of intervention needed.

Recruitment and the administration of the AUDIT-C tool took place across four sites. This included two primary healthcare clinics, Community HIV Testing Services, and an Orphans, Vulnerable Children and Youth Community-based Organisation (OVCY CBO).

The primary healthcare settings comprised the River Park and East Bank clinics. Both clinics provide primary healthcare services, in addition to other specialised services. Recruitment occurred in the primary care sections of both primary healthcare settings. The recruitment process was based on convenience sampling in both primary healthcare settings, and participants were not recruited based on their reasons for visiting the clinics. The recruitment process took place in the public communal areas of the primary health care settings, the screenings took place in cordoned-off health desk areas.

The Community HIV Testing Services, as well as the OVCY CBO, aligned with some of the interventions managed by HIVSA. Potential participants were then recruited through mobile testing services, home visits, and recruitment in informal community settings. The recruitment and screening processes across the mobile testing services and home visits took place in private settings.

### Referral sites

The River Park Clinic is a public primary healthcare facility that includes the River Park Community-based Substance Abuse Treatment Centre, which opened in 2018. The overall intent of the centre, as a division of the clinic, is to provide outpatient rehabilitation services to substance users and their families, and as such, the River Park Clinic was also nominated as a referral site for individuals identified by the AUDIT-C screening process as needing alcohol use counselling or treatment. The East Bank Clinic provides psychiatric and psychological services to individuals across different life stages, in addition to primary health care services, and was therefore also nominated as a referral site. The South African National Council on Alcoholism (SANCA) was also a referral partner, although not a screening site.

### Sample

Participant selection criteria included individuals aged 18 years or older who were living and/or working in Alexandra township during the research.

### Measure

This study implemented the AUDIT-C, a shortened version of the 10-question AUDIT instrument, comprising 3-item alcohol consumption questions. The AUDIT and AUDIT-C have been recognised as valid instruments to screen for possible unhealthy alcohol use [20, 21]. The AUDIT includes three domains relating to the level of alcohol consumption, evidence of dependence and harm from drinking. The shortened AUDIT-C is considered to perform as well as the full AUDIT in primary healthcare settings [21]. The AUDIT-C has also been implemented in different Southern African settings, at times indicating greater sensitivity to risk than other instruments [19, 20].

For the administration of the AUDIT-C tool, 12 g of pure alcohol was considered a standard drink, as typically defined in South Africa [22, 23]. Additionally, the imagery of a standard drink and the descriptive language were adjusted to ensure relevance to Alexandra township. This included references to 30 ml spirits as a ‘tot’ and a 330 ml beer as a ‘dumpie’. A South African-specific beverage, ‘traditional home-brewed beer called umcombotsi’, was also included. The alcohol percentage of traditional home-brewed beer/umcombotsi cannot be determined since it is subject to various home-brewing recipes [24]. The latter was particularly relevant during the COVID-19 pandemic, given the full and partial restrictions on alcohol sales.

The study protocol also required that the lay counsellors indicate whether brief advice or a referral was provided and whether the referral was accepted or declined. The respondents' age and sex were also recorded. Research suggests that the optimal cut-off score of the AUDIT-C may vary across contexts, populations and countries. The AUDIT-C score, as per AUDIT, therefore, allows for an adjustment of the screening threshold for particular settings. The scoring should therefore be influenced by national and cultural standards as assessed by clinicians [20]. This study implemented a score of 5 as the threshold for a positive AUDIT-C score because it has been shown to be an optimal cut-off score by select studies [21, 25, 33]. Additionally, this cut-off score has been found to be appropriately sensitive while also preventing high false-positive rates [21, 25]. The latter was considered of importance in the already resource-constrained context. Each AUDIT-C question is scored 0 to 4 points, resulting in a total score ranging from 0 to 12 [26]. A total score ranging from 0 to 4 is considered low-risk alcohol consumption, a score ranging from 5 to 7 is regarded as moderate risk and a score of 8 to 12 signals high risk alcohol consumption behavior. Participants with a score of 5 and above are recommended to receive brief advice, whereas both brief advice and a referral are recommended for participants with a score of 8 and above. In the current study, individuals scoring 1 to 4 also received a brief intervention as a preventive measure.

### Data entry and analysis

Data entry staff were employed to capture survey responses in Microsoft Excel. Data-cleaning processes included removing records where demographic or AUDIT-C-specific responses were missing or captured incorrectly.

Age was categorised to allow for a nonlinear association between age and the AUDIT-C score. The associations of gender, age (categorised) and AUDIT-C score were determined by the chi-squared test. The odds ratio (OR) of each study variable, rejection of brief advice and rejection of referral was determined using binomial regression. Reference categories were determined based on sample size and clinical relevance (place of screening: largest site; age: youngest age category; gender: female (because males were thought to be at higher risk of rejection of advice/referrals); AUDIT-C score: 5–8 for brief advice rejection (lowest AUDIT score for which brief advice is routinely given) and 8 for referral rejection (lowest AUDIT score for which referral is routinely given). Variables that were significant at the univariate level were included in a multivariable binomial regression analysis. Nonsignificant variables were sequentially removed from the multivariable model. Data analysis was carried out using STATA version 8. A 1% significance level was used.

## Results

The research reached a high percentage of the target population, while nonprobability and nonrandom sampling were utilised. A total of 60,022 participants were screened with the AUDIT-C measure. Of these, 54,187 AUDIT-C data collection tools were fully completed, without any apparent errors. This sample size represents close to 15% of all people aged 18 and over living and/or working in Alexandra township [19].

As noted in Table 1, 57% of participants were female, and 43% were male. Participants’ ages ranged from 18 to 85 years old, with a median age of 34. The age group with the highest representation included those aged between 31 and 39 (35.47%), and approximately 11% of participants were older than 50 years.

**Table 1:** Sample Characteristics

Characteristic	Category	n	%
Place of screening	Community programme (HIVSA)	28 131	51.91
	River Park Clinic	10 561	19.49
	East Bank Clinic	6 866	12.67
	Home visit	6 561	12.11
	Other	2 068	3.82
Gender	Female	31 019	57.24
	Male	23 168	42.76
Age (years)	18–24	7 757	14.32
	25–29	8 889	16.40
	30–39	19 221	35.47
	40–49	12 216	22.54
	50–59	4 457	8.23
	60–69	1 403	2.50
	70 and over	244	0.45
AUDIT-C total score (grouped)	0	6 438	11.88
	1 to 4	12 483	23.04
	5 to 7	23 283	42.97
	8 to 12	11 983	22.11

Regarding participant recruitment and distribution across the four research sites, most participants (*n* = 28,131 or 51.9%) were reached through the HIV Community Programme. ‘Other’ represents sites that were primarily informal gathering spaces in Alexandra township.

Related to participants’ AUDIT-C scores, 34.9% of participants recorded low scores, 43.0% recorded moderate scores, and 22.1% recorded high scores. Cumulatively, 65.1% of participants (*n* = 35,266) recorded moderate to high scores.

Gender and age significantly influenced AUDIT-C scores; 78% of male participants compared to 55.24% of female participants recorded moderate to high AUDIT-C scores. AUDIT-C scores increased with age.

### Factors that influenced the acceptance of brief advice and referrals

The analysis focused on participants who received a moderate score (AUDIT-C score of 5 or above) for the brief advice intervention and on participants who received high scores (AUDIT-C score of 8 +) on the AUDIT-C for the referral intervention. Table 2 highlights how brief advice was received with relatively high levels of acceptance by participants (95.2%). However, only 9.4% of participants who recorded high scores accepted referrals to local public health clinics or service providers. Participants’ age was positively correlated with the rejection of brief advice, and within the age categories.

**Table 2:** Acceptance of brief advice and referrals

Intervention	Response	n	%
Brief Advice	Accepted	45 902	95.21
	Rejected	2 310	4.79
	Not offered	5 975	-
Referral	Accepted	1 048	9.35
	Rejected	10 161	90.65
	Not offered	42 978	-

Multivariable analyses were utilised to identify factors that influenced participants’ rejection of brief advice and referral interventions. Table 3 presents the results for brief advice, while Table 4 presents the results for referrals.

**Table 3:** Results of multivariate analysis of factors affecting rejection of brief advice intervention

	Odds of rejection of brief advice		
	Odds ratio	[95% Conf	Interval]
Place of screening			
Community program [Reference]	1.00		
East Bank Clinic	0.95***	0.94	0.96
River Park Clinic	0.93***	0.93	0.94
Home Visit	0.90***	0.90	0.91
Other	0.89***	0.88	0.90
Gender			
Female [Reference]	1.00		
Male	1.01***	1.01	1.02
Age group			
18–24 [Reference]	1.00		
25–29	1.04***	1.03	1.05
30–39	1.04***	1.03	1.04
40–49	1.05***	1.04	1.05
50–59	1.11***	1.10	1.12
60–69	1.23***	1.21	1.25
70 and Over	1.29***	1.23	1.34
Audit score			
5–7 [Reference]	1.00		
8–12	1.16***	1.16	1.17
Log-likelihood			2381.9
AIC			-0.137
Number of observations			34,576

^***^ *p* < 0.001

**Table 4:** Results of multivariate analysis of factors affecting rejection of the referral intervention

	Odds of rejection of referral intervention		
	Odds ratio	[95% Conf	Interval]
Place of screening			
Community program [Reference]	1.00		
East Bank Clinic	1.02	0.98	1.07
River Park Clinic	1.16***	1.13	1.18
Home Visit	0.95***	0.93	0.96
Other	1.22***	1.18	1.26
Gender			
Female [Reference]	1.00		
Male	1.18***	1.16	1.20
Age group			
18–24 [Reference]	1.00		
25–29	2.16***	2.12	2.21
30–39	2.12***	2.08	2.15
40–49	2.12***	2.09	2.16
50–59	2.12***	2.08	2.17
60–69	2.11***	2.04	2.19
Over 70	2.17***	1.97	2.39
Audit—C score			
8 [Reference]	1.00		
9	1.10***	1.08	1.12
10	1.07***	1.05	1.09
11	1.05***	1.02	1.07
12	1.02	1.00	1.04
Log-likelihood			-3697.1
AIC			0.673
Number of observations			11,030

^***^ *p* < 0.001

Place of screening, gender, age and AUDIT-C scores all influenced the rejection of brief advice. Participants who were screened at ‘other’ locations (OR 0.89; 95% CI 0.88–0.90) were less likely to reject brief advice than those reached through the community programme, primary health care settings and home visits.

Men were more likely to reject brief advice than women (OR 1.01; 95% CI 1.01–1.02). Participants with high AUDIT-C scores were more likely to reject brief advice than those with moderate scores (OR 1.16; 95% CI 1.16–1.17). Finally, the odds of rejecting brief advice increased with age.

Similar to brief advice intervention, place of screening, participant gender, age and AUDIT-C scores affected participants’ rejection of referrals. The odds of rejection associated with referral rejection were noted for older participants and males (OR 1.18; 95% CI 1.16–1.20). The odds also increased with AUDIT-C scores and were higher for those screened at a place other than those listed (OR 1.22; 95% CI 1.18–1.26) and among those screened at the River Park clinic (OR 1.16; 95% CI 1.13–1.18).

## Discussion

The aim of this study was to investigate the use of the AUDIT-C as a source of referral for participants presenting patterns of high-risk alcohol consumption in a South African township. The findings suggest that the AUDIT-C is feasible in terms of administration across primary healthcare settings as well as community settings. This aligns with previous research that has demonstrated the AUDIT-C’s feasibility in primary healthcare settings [30, 32].

The findings suggest that the risk for rejection of referral is dependent on the extent of unhealthy alcohol use as measured by the AUDIT-C. Similarly, while most participants who scored 5 and below were open to receiving brief advice, resistance to brief advice increased with higher AUDIT-C scores. This shows the value of AUDIT-C in measuring unhealthy alcohol use. These findings are supported by previous research that suggests that individuals with unhealthy alcohol use often do not seek intervention as they may not consider their behaviour problematic, and/or may not wish to stop consuming alcohol [27, 30]. In some cases, resistance to seeking treatment may be a feature of alcohol use disorder [28, 29].

The results also indicated that men were most likely to report patterns of high-risk alcohol consumption. This aligns with the WHO’s [2] findings that identify men as the gender that comparatively consumes more alcohol. While the difference between men and women was not great in magnitude, it may be in keeping with research findings specific to the South African context that demonstrate that binge drinking is most prevalent among men [31, 32]. This study’s findings indicate that men were also statistically more likely to reject an outpatient referral. These findings may be understood in relation to research pertaining to men’s general lack of health-seeking behaviour. This highlights concerns about toxic masculinity and stigma [31, 34, 35]. This difficulty with general help-seeking behaviour may explain why men struggle to accept brief advice or referrals. Moreover, more rigid masculine ideals may have made it difficult for men to accept brief advice or referrals related to alcohol consumption from lay counsellors, who were predominantly women. While gendered patterns of alcohol consumption have been noted globally [2, 25], importantly, intersections of gender, race, and alcohol consumption in South Africa also contribute to toxic masculine traits, such as ‘real men’ being those who can consume and handle large amounts of alcohol [6, 26]. This may in turn influence self-reporting among men, given that drinking much alcohol may be equated to manhood.

Furthermore, the results suggest that while participants were relatively open to receiving brief advice, they were often reluctant to take active steps in accepting a referral to treatment to reduce their alcohol consumption. This may be attributed to various factors, including stigma and resource constraints impacting time and transport, particularly in low-resource settings. It is therefore important that effective and holistic measures are taken to facilitate a reduction in alcohol use in these communities. Certainly, this must include, among others, an effective screening, brief advice and referral process in response to the current levels of observed resistance and to the systemic variables that drive unhealthy alcohol use.

The digital transformation of health systems, particularly in low- and middle-income countries, could offer an opportunity to expand population access cost-effectively and cost-efficiently for preventative health interventions such as AUDIT C as a screening tool for unhealthy alcohol use. Moreover, the advantage of digital channels of administration includes enhanced privacy and confidentiality, which could increase intervention uptake, as has been demonstrated by other healthcare programs such as HIV/AIDS and Tuberculosis prevention [36].

### Limitations and strengths

The findings should be reviewed in relation to the study’s limitations and strengths. The study presented various limitations. A limitation of this research is that it was an uncontrolled study. Additionally, no other measures were included in the study to provide a lens of comparison. Future research may consider including the AUDIT or other shortened versions of the AUDIT. A further limitation is that the screening and brief intervention was conducted by lay counsellors with varied levels of experience, and it cannot be assessed how their behaviours may have influenced responses. Moreover, while training comprised different components and phases, additional booster sessions may have addressed the issue pertaining to experience. An important consideration is that privacy could not be assured across all settings, and it is not known how this may have influenced the level of response.

Despite these limitations, this study also had several strengths. A strength of the study is the large study population, as well as adequate representation across age and gender. A further strength is that the research was administered across various settings, which facilitated an understanding of the administration of the AUDIT-C across both primary healthcare settings and community settings.

Research related to screening and brief interventions has received considerable attention in the Global North and therefore mostly in high-income settings. This study contributes to the knowledge base of screening and brief interventions in Africa and the greater Global South.

## Conclusion

Unhealthy alcohol use contributes significantly to premature death and disability globally while also having detrimental effects on societal well-being. Rates of alcohol consumption vary significantly by region, with South Africa evidencing continued unhealthy alcohol use. Despite the latter, South Africa continues to observe alcohol industry interference [10, 11], hindering its progress towards effective harm reduction policies. The lack of effective policies has rendered the country more dependent on intervention strategies.

This research initiative evaluated the use of screening with AUDIT-C, combined with brief intervention, and potential referral, in the resource-constrained Alexandra township. The findings demonstrate the feasibility of administering the AUDIT-C across a range of primary health care and community settings. However, resistance to brief advice increases with higher AUDIT-C scores, along with resistance to referrals. AUDIT-C is likely a useful tool for detecting unhealthy alcohol use and prompting conversations about drinking in other low-resource settings.

## Data Availability

The data that support the findings of this study are available from the ABInBev Foundation, but restrictions apply to the availability of the data. Data are however available from the authors upon reasonable request and with permission of the ABInBev Foundation. The corresponding author can be contacted should someone want to request the data from this study.

## Abbreviations

AUDIT-C: Alcohol Use Disorders Identification Test–Consumption
HIVSA: HIV South Africa
OVCY CBO: Orphans, vulnerable children and youth community-based organisation
SANCA: South African National Council on Alcoholism
